# Correction: Bouzayan et al. Assessment of Mental Health Comorbidities and Relief Factors in Moroccan Women During the Third Trimester of Pregnancy: A Cross-Sectional Study. *Healthcare* 2024, *12*, 1470

**DOI:** 10.3390/healthcare14183021

**Published:** 2026-09-15

**Authors:** Ranya Bouzayan, Maroua Guerroumi, Amina Aquil, Noura Dahbi, Ouassil El Kherchi, Salma Ait Bouighoulidne, Soumia Ait Ami, Meryam Belhaj Haddou, Arumugam R. Jayakumar, Abdeljalil Elgot

**Affiliations:** 1 Epidemiology and Biomedical Unit, Laboratory of Medical Biotechnology (Med Biotech), Faculty of Medicine and Pharmacy, Mohammed V University, Rabat 10000, Morocco; ranya_bouzayan@um5.ac.ma; 2 Epidemiology and Biomedical Unit, Laboratory of Sciences and Health Technologies, Higher Institute of Health Sciences, Hassan First University of Settat, Settat 26000, Morocco; m.guerroumi@uhp.ac.ma (M.G.); amina.aquil@uhp.ac.ma (A.A.); no.dahbi@uhp.ac.ma (N.D.); e.ouassil@uhp.ac.ma (O.E.K.); s.aitbouighoulidne@uhp.ac.ma (S.A.B.); 3 University Teaching Hospital Mohammed VI, Marrakech 40070, Morocco; s.aitami.res@uca.ac.ma (S.A.A.); m.belhajhaddou@uhp.ac.ma (M.B.H.); 4 Department of Obstetrics, Gynecology & Reproductive Sciences, Miller School of Medicine, University of Miami, Miami, FL 33136, USA; ajayakumar@med.miami.edu

## Addition of an Author

Ranya Bouzayan was not included as an author in the original publication [1]. The corrected Author Contributions statement appears here:

**Author Contributions:** Conceptualization, A.E., R.B. and M.G.; methodology, A.A., R.B. and M.G.; validation, A.E. and A.A.; formal analysis, R.B., M.G., N.D. and M.B.H.; investigation, R.B. and M.G.; data curation, O.E.K., R.B., M.G. and M.B.H.; writing—original draft preparation, R.B., M.G., S.A.B. and A.A.; writing—review and editing, R.B., M.G., O.E.K. and A.E.; visualization, S.A.A. and S.A.B.; supervision, A.E. and A.R.J.; funding acquisition, R.B. and M.G. All authors have read and agreed to the published version of the manuscript.

There was also an error regarding the affiliation for Soumia Ait Ami. The updated affiliation should be affiliation 3.

The authors state that the scientific conclusions are unaffected. This correction was approved by the Academic Editor. The original publication has also been updated.
